# Poor Thermal Care Practices among Home Births in Nepal: Further Analysis of Nepal Demographic and Health Survey 2011

**DOI:** 10.1371/journal.pone.0089950

**Published:** 2014-02-28

**Authors:** Vishnu Khanal, Tania Gavidia, Mandira Adhikari, Shiva Raj Mishra, Rajendra Karkee

**Affiliations:** 1 School of Public Health, Curtin University, Perth, Australia; 2 Centre for International Health, Curtin University, Perth, Australia; 3 Public Health Worker, Kathmandu, Nepal; 4 Institute of Medicine, Kathmandu, Nepal; 5 School of Public Health and Community Medicine, BP Koirala Institute of Health Sciences, Dharan, Nepal; University of Alabama at Birmingham, United States of America

## Abstract

**Introduction:**

Hypothermia is a major factor associated with neonatal mortality in low and middle income countries. Thermal care protection of newborn through a series of measures taken at birth and during the initial days of life is recommended to reduce the hypothermia and associated neonatal mortality. This study aimed to identify the prevalence of and the factors associated with receiving ‘optimum thermal care’ among home born newborns of Nepal.

**Methods:**

Data from the Nepal Demographic and Health Surveys (NDHS) 2011 were used for this study. Women who reported a home birth for their most recent childbirth was included in the study. Factors associated with optimum thermal care were examined using Chi-square test followed by logistic regression.

**Results:**

A total of 2464 newborns were included in the study. A total of 57.6 % were dried before the placenta was delivered; 60.3% were wrapped; 24.5% had not bathing during the first 24 hours, and 63.9% were breastfed within one hour of birth. Overall, only 248 (10.7%; 95% CI (8.8 %, 12.9%)) newborns received optimum thermal care. Newborns whose mothers had achieved higher education (OR 2.810; 95% CI (1.132, 6.976)), attended four or more antenatal care visits (OR 2.563; 95% CI (1.309, 5.017)), and those whose birth were attended by skilled attendants (OR 2.178; 95% CI (1.428, 3.323)) were likely to receive optimum thermal care.

**Conclusion:**

The current study showed that only one in ten newborns in Nepal received optimum thermal care. Future newborn survival programs should focus on those mothers who are uneducated; who do not attend the recommended four or more attend antenatal care visits; and those who deliver without the assistance of skilled birth attendants to reduce the risk of neonatal hypothermia in Nepal.

## Introduction

Neonatal hypothermia, defined as a body temperature less than 36.5 degree Celsius, is widely recognised as an important contributing factor to neonatal morbidity, in particular in low and middle income countries [1]–[4]. Unlike adults and children, newborns have a limited capacity to maintain an optimal core body temperature. The smaller and more premature the baby, the more vulnerable they are to developing neonatal hypothermia. Unless heat loss is prevented in the period immediately after birth, a healthy term baby will lose on average 0.1 to 0.3 degree Celsius per minute [5]. This phenomenon takes place by conduction, convection, evaporation and radiation from the body of newborn [6]. Such temperature drop can lead to a variety of adverse consequences such as hypoglycaemia, respiratory distress, hypoxia, metabolic acidosis, and even death [5]. To protect a newborn from such consequences of hypothermia, appropriate external thermal protective measures are essential [5].

Inadequate thermal care increases the risk of hypothermia, especially in low birth weight children. In response, the World Health Organization (WHO) recommends thermal protection during the initial days of life. Success in adapting such practices is measured as the proportion of women and newborns practicing these life-saving behaviours [7].

A number of studies in developing countries have shown that the neonates are at risk of suffering hypothermia. A recent global review [8] has reported a prevalence of hypothermia between 32 % – 85% among hospital deliveries and 11%–92% among home deliveries. Other authors also report that hypothermia prevalence was higher among babies delivered at home compared to hospital settings [9]–[11]. For Nepal, such estimates are limited, however a recent study of neonatal hypothermia in southern Nepal, found that 92% of babies were hypothermic during the first 28 days of life, irrespective of place of birth [10]. Despite a large number of studies in newborn care from developing countries, limited data are available on how thermal care is provided to the newborns of those countries; and what proportion of newborns receive the recommended thermal care.

Nepal has made considerable progress in achieving Millennium Development Goal (MDG) 4 for child survival. The under-five mortality has reduced from 162 per 1,000 live births in 1990 to 54 in 2011 [12], and infant mortality was reduced from 108 to 46 per 1000 live births in the same period. Between 2001 and 2006, the neonatal mortality rate dropped from 43 to 22 per 1,000 live births and did not decline any further between 2006–2011. Neonatal mortality accounts for approximately 60% of deaths of children under the age of five in Nepal [13], suggesting that reaching the MDG neonatal mortality target of 16 per 1,000 live births for 2015 is going to require a substantial reduction in newborn mortality [12]. While addressing neonatal hypothermia may help achieve the overall MDG for child survival, it has so far been a neglected challenge [12].To accelerate the decline in neonatal and child mortality associated with hypothermia in Nepal, the Ministry of Health and Population (MoHP) [14] of Nepal based on the WHO newborn care guidelines and local intervention study [15]has recommended the following thermal care practices: (1) wiping the newborn with a soft, dry cloth immediately after birth; (2) putting the newborn on the mother’s chest and initiating skin-to-skin contact(Kangaroo mother care for low birth weight newborns); (3) providing advice on the initiation of breastfeeding within one hour of birth; (4) wrapping the newborn immediately after birth; and (5) bathing the newborn only after 24 hours post-birth along with other measures such as prioritized skill attendance at birth, use of safe delivery kits, and cord care [16], [17]. Because prevention and treating newborn hypothermia in health institutions and community setting is relatively easy and cost effective, it is important that the recommended practices to preventing hypothermia are universally adopted. To date, a number of studies have reported on the newborn care practices in Nepal [3], [15], [18], [19], however, no study has reported to what extent Nepalese mothers are meeting the recommended thermal care practices. A large study (N = 22941) [20] from Southern Nepal reported a universal rate of bathing of newborns soon after birth (99.3%), and massage with mustard oil (99.8%). Both of these traditional newborn practices are likely to subject a newborn to hypothermia due to heat loss from body. The risk of hypothermia may be even more in the mountainous part of the Nepal which covers two thirds of the country.

The high burden of low birth weight (LBW) experienced in Nepal, estimated to range between 12%–50% [21], [22] is likely to be contributing to the high prevalence of neonatal hypothermia reported in earlier studies [23] as LBW babies decreased stores of brown fat and glycogen, and may not be able to conserve or generate body heat [5]. In addition, Nepali mothers demonstrate poor uptake of recommended practices such as skin to skin contact of newborns, whereby only 4.5% of mothers surveyed in a community based study of Southern Nepal reported skin to skin contact [20].Furthermore, the high prevalence of home births experienced in Nepal (65%) [22] and the lack of professional assistance during home births, is likely to conserve traditional newborn care practices which increase the risk of neonatal hypothermia, while in turn reducing the likelihood of a mother performing all the recommended thermal care practices.

It is this inability to abide by the recommended practices increases the risk of neonatal hypothermia and the associated mortality. Therefore, this study aimed at identifying the proportion of newborns receiving optimum thermal care and the factors associated with optimum thermal care. Optimum thermal care in this study refers four thermal care practices for which data was available in the Nepal Demographic and Health Survey (NDHS) 2011.

## Methods

### Study population

This study used the dataset from Nepal Demographic and Health Survey (NDHS) 2011 [22]. The NDHS is a cross sectional study conducted every five years. The 2011 NDHS surveyed 5,306 mother-child pairs of which, 2464 children were the last born children born in home in the last five years. The response rate for household was 99.4% and overall women response rate was 97.6%. Information on non-institutional newborn thermal care practices was collected in the NDHS 2011. The details of sampling and methodology of the NDHS 2011 is provided in respective reports (available from: http://www.measuredhs.com/publications/publication-fr257-dhs-final-reports.cfm) [22].

A total of 13 domains were included in the NDHS 2011 and from which 25 strata were created as rural and urban locations [22]. From each domain, samples were selected based on two stage sampling methods. In the first stage, enumeration areas (clusters) were selected using probability proportionate to size. In the second stage, 35 households in urban areas and 40 households in rural areas were selected based on systematic random sampling from each enumeration areas [22]. Sample weights were allocated to the samples due to such unequal sample collection from rural and urban areas.

### Variables

In NDHS 2011, information on thermal care was collected for home births. These practices are complementary to each other and therefore it is expected that all of these care should be provided to a newborn. The outcome variable assessed in this study was “optimum thermal care” defined as the proportion of mothers who reported providing four thermal care practices : (i) drying the newborn immediately after birth, (ii) wrapping the child before placenta is delivered; (iii) delaying bathing of newborn for 24 hours; and (iv) initiation of breastfeeding within the first hour of birth. The definition of optimum care has been used in a previous study [24].While other thermal care practices are important for newborn survival, due to the unavailability of data on all thermal care practices in the NDHS 2011, this study is restricted to the four practices mentioned above.

The independent variables used in this study were drawn from previously published literature on newborn health [20], [25]–[27]. The independent variables were grouped as predisposing, enabling and external factors [26]. Predisposing factors such as maternal age, parental education, maternal occupation, ethnicity, religion, wealth status, sex of child, birth order, and perceived size of child affect newborn care practices indirectly by influencing on mothers decision making on care of a newborn. Enabling factors such as attending antenatal care and having a skilled attendant during delivery influence thermal care practices by influencing and facilitating a mother to adopt the recommended newborn practices. On the other hand, external factors such as rural/urban residence, development regions and ecological regions influence newborn care practices by influencing access to information, and services. Recoding of the variables were adopted from previously published DHS based studies [26], [27].

### Statistical analysis

Optimum thermal care was reported as proportion and their 95% confidence intervals (CI). The independent variables associated with optimum thermal care were examined by using Chi-square test. The significant factors were then further examined using logistic regression. Stepwise backward elimination process was used during multiple logistic regression to investigate factors associated with optimum thermal care. A p-value <0.05 is set statistically significant. Complex Sample Analysis method was used to account for the study design and sample weight [27], [28].This method allows to account for strata, cluster and sample weights during analyses. Considering such weights during analyses gives a more precise point estimation and the measure of association [29].

### Ethics statement

The DHS surveys were approved by Nepal Health Research Council, Nepal and ICF Macro Institutional Review Board in Calverton, Maryland, USA. Permission from Macro International (the research agency) was obtained for use of the data [22]. Mothers, the household and the men included in the study provided written consent (or thumb print) for the study. Care takers or the mothers provided the consent for the children included in the survey. The data was stored in the database of ICF Macro international, Measure DHS program [30] .The current dataset was made available for public use after removing the personal identifiers.

## Results

### Practice of optimum thermal care

Table 1describes the thermal care practices. Of total 2464 newborns, 57.6 % were dried before placenta delivered; 60.3% were wrapped; 24.5% had no bathing during the first 24 hours; and 63.9% were breastfed within one hour of birth. Overall, 575 (24.1 %) newborns received only one recommended thermal care practice, 562 (23.4%) received two, while 836 (30.5%) received three thermal care practices. Only 248 (10.7%; 95% CI (8.8 % –12.9%)) received all four recommended thermal care practices i.e. optimum thermal care. A significant proportion (n = 243, 11.3 %) of newborns did not receive any recommended thermal care practices.

**Table 1:** Thermal care practices among Nepalese Mothers (N =  2464).

	Thermal Care Practices	Number	Per cent	95% Proportion
1	Child dried before placenta was delivered	1461	57.6	53.8–61.4
2	Child was wrapped in cloth before placenta was delivered	1555	60.3	56.3–64.2
3	No bathing during the first 24 hours	529	24.5	21.4–28.0
4	Initiation of breastfeeding within one hour	1670	63.9	59.6–67.9
	**Optimum thermal Care (1,2,3, and 4)**	**248**	**10.7**	**8.8**–**12.9**

### Characteristics of the participants

Table 2 describes the characteristics of the participants. Of the 2464 children, 539 (21.9%) were in 0–11 months age group, 549 (22.3%) in 12–23, 512 (20.8%) in 24–35, 498 (20.2%) in 36–47, and 366 (14.9%) were in 48–59 months age group. The majority of mothers were from the age group 20–29 years (60.5%). More than a half did not have formal education (57.3%). The majority (65.1%) were from poor households. Only 36.3% of the mothers reported attending the recommended four or more ANC visits, and only 9.3% of home deliveries were attended by a skilled person.

**Table 2:** Proportion (%) optimum thermal care among the children born in home, Nepal 2011 (N = 2464).

Factor	Total N [%]#	Provided optimum thermal care	P value
**Predisposing factors**			
**Mother’s age**			0.008
15–19	135 (5.5)	16 (10.1)	
20–29	1490 (60.5)	172 (12.1)	
30–34	435 (17.7)	43 (11.0)	
> = 35	404 (16.4)	17 (4.8)	
**Maternal Education**			<0.001
No education	1412 (57.3)	96 (7.4)	
Primary	515 (20.9)	42 (8.1)	
Secondary	497 (20.2)	100 (21.4)	
Higher	40 (1.6)	10 (26.1)	
**Mother’s occupation**			0.050
Not working	372 (15.1)	47 (13.7)	
Agriculture	1859 (75.4)	160 (9.3)	
Working (paid)	233 (9.5)	41 (14.7)	
**Father’s Education**			<0.001
No education	619 (25.1)	43 (6.7)	
Primary	719 (29.2)	57 (8.9)	
Secondary	978 (39.7)	115 (13.3)	
Higher	148 (6.0)	33 (23.6)	
**Ethnicity**			0.050
Relatively advantaged	1060 (43.0)	100 (10.1)	
Relatively disadvantaged (Janjati)	933 (37.9)	106 (11.5)	
Relatively disadvantaged (Dalit)	471 (19.1)	42 (17.7)	
**Religion**			0.331
Hindu	2062 (83.7)	203 (11.1)	
Others	402 (16.3)	45 (8.9)	
**Wealth status**			<0.001
Poor (Lower 40%)	1603 (65.1)	110 (7.5)	
Middle (Middle 40%)	739 (30.0)	116 (14.2)	
Rich (Upper 20%)	122 (5.0)	22 (23.4)	
**Sex of child**			0.492
Male	1331 (54.0)	136 (10.2)	
Female	1133 (46.0)	112 (11.2)	
**Birth order**			0.006
First	569 (23.1)	66 (13.0)	
Second or third	993 (40.3)	131 (12.2)	
Fourth or more	902 (36.6)	51 (6.8)	
**Size of baby**			0.506
Large	458 (18.6)	49 (11.5)	
Average	1514 (61.5)	160 (11.0)	
Small	490 (19.9)	39 (8.7)	
**Enabling factors**			
**ANC visit (Times)**			<0.001
No ANC visit	569 (23.1)	25 (4.7)	
1–3	993 (40.3)	85 (9.1)	
4 or more	902 (36.3)	138 (16.5)	
**Skilled attendance of home delivery**			<0.001
No Skilled	2234 (90.7)	184 (9.1)	
Skilled	230 (9.3)	64 (24.0)	
**External Environment**al **Factors**			
**Place of residence**			0.275
Urban	280 (11.4)	41 (13.3)	
Rural	2184 (88.6)	207 (10.6)	
**Development region**			0.119
Eastern	579 (23.5)	72 (13.0)	
Central	491 (19.9)	36 (8.4)	
Western	334 (13.6)	46 (14.2)	
Mid -Western	585 (23.7)	43 (7.1)	
Far-Western	475 (19.3)	51 (11.4)	
**Ecological region**			0.113
Mountain	576 (23.4)	45 (7.0)	
Hill	1051 (42.7)	95 (9.6)	
Terai/Plain	837 (34.0)	108 (12.3)	

The percentages presented for the thermal care are the weighted and cluster sampling adjusted percentage which differs from the crude percentage. The proportion of thermal care practice in each category are the row percent. The number of missing values may vary for each variable. # the number and percent reported are unweighted for the independent variables.

### Factors associated with providing optimum thermal care

Table 2 also shows the thermal care practices of the study participants by their characteristics. It should be noted that 24.0 % of deliveries where skilled attendant present have received the optimum thermal care. A small proportion (8.7%) of higher order (4 or more) infants received optimum thermal care. Table 3 presents the results of the unadjusted and adjusted odds ratios of receiving optimum thermal care obtained using logistic regression models. After controlling for other socio-demographic factors, maternal education, the number of antenatal care visits and having a skilled attendant during delivery remained statistically significant. The infants who were born to educated mother such as having higher education (OR 2.8100; 95% CI (1.132, 6.976)), and secondary education (OR 2.511; 95% CI (1.623, 3.887)) were more likely to receive optimum thermal care. Attending the recommended four ANC visits was associated with higher odds (OR 2.563; 95% CI (1.309, 5.017)) of receiving optimum thermal care than those counterparts whose mothers did not have any ANC visit. Furthermore, having skilled attendant (OR 2.178; 95% CI (1.428, 3.323)) during delivery was significantly associated with receiving optimum thermal care.

**Table 3:** Factor associated with optimum thermal care among home deliveries of Nepal, NDHS 2011.

Factor	Unadjusted OR (95 % CI)	Adjusted OR (95 % CI)
**Mother’s age at pregnancy**	P = 0.010	P = 0.269
15–19	1.00	1.00
20–29	1.226 (0.626, 2.403)	1.597 (0.816, 3.128)
30–34	1.101 (0.518, 2.340)	1.734 (0.817, 3.679)
> = 35	0.448 (0.184, 1.088)	1.089 (0.420, 2.824)
**Maternal education**	P<0.001	P<0.001
No education	1.00	1.00
Primary	1.104 (0.696, 1.750)	0.938 (0.581, 1.512)
Secondary	3.432 (2.293, 5.136)	2.511 (1.623, 3.887)
Higher	4.433 (2.048, 9.598)	2.810 (1.132, 6.976)
**Mother’s occupation**	P = 0.032	P = 0.579
Not working	1.00	1.00
Agriculture	0.649 (0.410, 1.025)	0.781 (0.485, 1.258)
Working (paid)	1.089 (0.570, 2.080)	0.793 (0.408, 1.543)
**Father’s Education**	P<0.001	P = 0.605
No education	1.00	1.00
Primary	1.367 (0.827, 2.261)	1.140 (0.664, 1.957)
Secondary	2.143 (1.347, 3.409)	1.166 (0.672, 2.025)
Higher	4.321 (2.223, 8.387)	1.752 (0.752, 4.078)
**Ethnicity**	P = 0.747	P = 0.079
Relatively advantaged	1.00	1.00
Relatively disadvantaged (Janjati)	1.169 (0.746, 1.789)	1.564 (1.001, 2.444)
Relatively disadvantaged (Dalit)	0.997 (0.554, 1.793)	1.631 (0.922, 2.885)
**Birth order**	P = 0.014	P = 0.629
First	1.00	1.00
Second or third	0.930 (0.643, 1.346)	1.235 (0.785, 1.945)
Fourth or more	0.490 ( 0.290, 0.813)	1.288 (0.675, 2.460)
**Wealth status**	P<0.001	P = 0.147
Poor (Lower 40%)	1.00	1.00
Middle (Middle 40%)	2.046 (1.408, 2.975)	1.309 (0.823, 2.081)
Rich (Upper 20%)	3.779 (1.844, 7.743)	1.522 (0.996, 2.327)
**ANC**	P<0.001	P = 0.008
No ANC visit	1.00	1.00
1–3	2.024 (1.103, 3.713)	1.740 (0.928, 3.265)
4 or more	3.898 (2.181, 7.296)	2.563 (1.309, 5.017)
**Skilled attendance in home delivery**	P<0.001	P = <0.001
No Skilled	1.00	1.00
Skilled	3.159 (2.155, 4.631)	2.178 (1.428, 3.323)

Hosmer and Lemeshow Goodness of Fit test: p = 0.981; Nagelkerke pseudo R square: 0.100.

## Discussion

This study has found that newborns in Nepal continue to be exposed to unfavourable practices immediately after birth, putting them at risk of neonatal hypothermia. In this study, approximately two in five neonates were not dried before the placenta was delivered; and three in four children were bathed within 24 hours. Overall, only 10.7 % of neonates received optimum thermal care as defined in this study. Previous study reported from Sarlahi district of Southern Nepal reported a very high rate (92%) of hypothermia [10]. The authors suggested that although the families kept the room warm, the practice of early bathing and massaging with oil reduced the benefit of such space heating. Similar to this study, previous studies have also found that the practice of bathing newborns within the first 24 hours after birth is a common practice in Nepal [19] and other developing countries [31]. The reasons cited for this practice were “cleaning of ritual pollution” due to childbirth and “child should be cleaned before being breastfed” [31]. Newborn practices influenced by cultural belief systems have also been reported in Bangladesh, where Darmstadt et al. [32] reported that newborn babies from Hindu families were bathed immediately after birth, including during the winter months, to make the newborn ‘holy’.

Wrapping in cloth prevents rapid heat loss from newborns. In this study, less than two thirds of newborn were wrapped in dry cloth. A previous study from Bangladesh [32] reported that the families in the home deliveries only use a small piece of cloth to wrap the newborn. Such insufficient wrapping increases the risk of hypothermia.

Overall, results from this study suggest that almost 90 % of children did not receive optimum thermal care. Given that Nepal has a high burden of neonatal mortality which since 1996 has decreased at a slower rate than the under-five mortality rate [13], suggests that poor newborn practices, including neonatal thermal care practices, may be contributing to the slow national decline with regard to neonatal mortality [3].

Attending the recommended ANC visits, having a skilled attendant during deliveries, and having an educated mother were associated with the optimum thermal care. The effect of attending ANC and having skilled attendant at birth are expected [25]. Health workers who provide antenatal care and delivery services are trained on essential newborn care. The newborn survival strategy in Nepal includes recommendations for births to be attended by trained health workers. The existing “Aama (means mother) program” in Nepal, which provides free delivery services and also cash incentives to mothers who deliver at certified health facilities, is also likely to influence thermal care positively, as the deliveries in health facilities are attended by skilled attendants [33]. As part of community based newborn care program in Nepal, the female community health volunteers are trained to keep the newborn warm and clean to reduce the mortality associated with hypothermia and sub-optimum newborn care [34], [35]. However, evaluation of this intervention on reducing hypothermia and any evidence on improved thermal care are yet to be reported.

Education of mothers has been reported to positively influence newborn health in Nepal, and elsewhere [27], [36]. The positive influence of mother’s education on optimum thermal care was also shown in our study. There are many indirect pathways that education may influence; for instance, educated mothers are more likely to use skilled attendant at birth, attend ANC [37], attend postnatal care [38] and understand the message provided by health workers and other media.

This study also found a difference in thermal care practice between advantaged and disadvantaged Janjati groups. Although the difference between advantaged groups and disadvantaged Dalits was marginal, further studies are needed to explore reasons why such difference exists. Contrary to the findings in this study, it is well established that advantaged groups have higher education level, generally better access to services and other opportunities than Dalit and Janjati groups [12]. This finding could be a reflection of the government’s increased focus on the poor and disadvantaged ethnicities with regards to maternal and child health as outlined in the two Three-Year Interim Plans (2006/07–2009/10 and2010/11–2013/14) [12]. Nevertheless, the results highlight the need to focus on advantaged ethnic groups to increase newborn thermal care practices.

This study used the national dataset which covered the entire country. The analysis accounted for the sampling design and cluster effect which provides a more precise point and interval estimates [28]. In a setting such as Nepal, where the majority of births occur at home, this study provides useful information for community based newborn survival programs. Recall bias is a pertinent issue in the dataset of the NDHS as it includes the information five years preceding the survey. However, usefulness of this dataset for neonatal health has been demonstrated in previous studies [26], [39]. The major limitation of this study is including only four major thermal care practices while other practices such as keeping house warm, giving skin to skin care for low birth weight newborns are also important thermal care practices. However, further thermal care information such as ambient temperature, practice of heating rooms, skin to skin care of low birth weight newborn, massaging the newborn are not available in the NDHS 2011. Previous studies have also reported a high prevalence of hypothermia in health facility deliveries in Nepal which need further examination on the specific practices related to health facilities. Future studies may consider examining more thermal care practices and their contribution to increase hypothermia. The effect of current newborn care community based interventions on increasing recommended thermal care practices is also need to be examined to ensure if these community based interventions are effective to reduce hypothermia and associated mortalities.

## Conclusion

This study found that only one in every ten home-delivered newborns was receiving optimum thermal care in Nepal. The newborns who were born to less educated mothers, born to mothers who did not attend any ANC visits, and whose birth were not attended by skilled workers were vulnerable to not receiving optimum thermal care. Newborn care program in Nepal should focus on these groups. Further investigation of thermal care practices with wider range of care practices and their contribution to neonatal mortality in Nepal is recommended.
